# Anxiety and Depression Rates in the Breast Cancer Population: A Multistudy Analysis

**DOI:** 10.1192/j.eurpsy.2026.11556

**Published:** 2026-07-31

**Authors:** M. Chelidze

**Affiliations:** Psychiatry, Todua clinic, Tbilisi, Georgia

## Abstract

**Introduction:**

Breast cancer remains one of the most prevalent malignancies affecting women globally, with millions diagnosed each year. While medical advancements have significantly improved survival rates, the psychological burden of breast cancer remains substantial. Anxiety and depression are among the most common mental health disorders experienced by breast cancer patients, triggered by the uncertainty of diagnosis, invasive treatments, body image changes, and fear of recurrence. Unfortunately, anxiety and depression are frequently underdiagnosed and insufficiently addressed in clinical oncology practice, leading to poorer health outcomes.

**Objectives:**

- Determine the rates at which anxiety and depression occur within the breast cancer patient population.
- Explore the demographic and clinical characteristics that are associated with increased psychological distress.
- Provide a comparative analysis of anxiety and depression prevalence across a selection of recent studies.

**Methods:**

A systematic review was performed. Used instruments were Hospital Anxiety and Depression Scale and the Patient Health Questionnaire-9. Relevant data including sample sizes, prevalence rates, and patient demographics were collected and organized. The analysis focused on assessing anxiety and depression rates in relation to factors such as patient age, cancer stage, treatment modalities, and levels of social support.

**Results:**

The prevalence of anxiety among breast cancer patients ranged from 28% to 45%, while depression rates varied from 22% to 33%. Elevated psychological distress was most notable during diagnosis and active treatment phases, especially chemotherapy.

**Conclusions:**

Anxiety and depression are common psychological conditions affecting individuals with breast cancer, significantly influencing their overall health and quality of life. Early and ongoing mental health screening should be an integral part of cancer care protocols. Incorporating a range of psychological support services, such as counseling, cognitive-behavioral therapy, and medication management, can effectively address these challenges.

**Disclosure of Interest:**

None Declared

